# Phospholipids are A Potentially Important Source of Tissue Biomarkers for Hepatocellular Carcinoma: Results of a Pilot Study Involving Targeted Metabolomics

**DOI:** 10.3390/diagnostics9040167

**Published:** 2019-10-29

**Authors:** Erin B. Evangelista, Sandi A. Kwee, Miles M. Sato, Lu Wang, Christoph Rettenmeier, Guoxiang Xie, Wei Jia, Linda L. Wong

**Affiliations:** 1 The Queen’s Medical Center, Honolulu, HI 96813, USA; erinbevangelista1998@gmail.com (E.B.E.); msato@queens.org (M.M.S.); 2 University of Hawaii Cancer Center, University of Hawaii at Manoa, Honolulu, HI 96813, USA; lwang@cc.hawaii.edu (L.W.); gxie@cc.hawaii.edu (G.X.); wjia@cc.hawaii.edu (W.J.); hepatoma@aol.com (L.L.W.); 3 Departments of Medicine and Surgery, John A. Burns School of Medicine, University of Hawaii, Honolulu, HI 96813, USA; crettenm@hawaii.edu

**Keywords:** hepatocellular carcinoma, metabolomics, diagnosis, phospholipids, machine learning, molecular imaging, positron emission tomography

## Abstract

Background: Hepatocellular carcinoma (HCC) pathogenesis involves the alteration of multiple liver-specific metabolic pathways. We systematically profiled cancer- and liver-related classes of metabolites in HCC and adjacent liver tissues and applied supervised machine learning to compare their potential yield for HCC biomarkers. Methods: Tumor and corresponding liver tissue samples were profiled as follows: Bile acids by ultra-performance liquid chromatography (LC) coupled to tandem mass spectrometry (MS), phospholipids by LC-MS/MS, and other small molecules including free fatty acids by gas chromatography—time of flight MS. The overall classification performance of metabolomic signatures derived by support vector machine (SVM) and random forests machine learning algorithms was then compared across classes of metabolite. Results: For each metabolite class, there was a plateau in classification performance with signatures of 10 metabolites. Phospholipid signatures consistently showed the highest discrimination for HCC followed by signatures derived from small molecules, free fatty acids, and bile acids with area under the receiver operating characteristic curve (AUC) values of 0.963, 0.934, 0.895, 0.695, respectively, for SVM-generated signatures comprised of 10 metabolites. Similar classification performance patterns were observed with signatures derived by random forests. Conclusion: Membrane phospholipids are a promising source of tissue biomarkers for discriminating between HCC tumor and liver tissue.

## 1. Introduction

Liver cancer is the fifth-most common cancer, and third-leading cause of cancer-related deaths worldwide, with over 90% of primary liver cancers being hepatocellular carcinoma (HCC) [1]. Guidelines by the National Comprehensive Cancer Network (NCCN) and American Association for the Study of Liver Disease (AASLD) allow for the diagnosis of HCC to be secured radiographically using contrast-enhanced computed tomography (CT) or magnetic resonance imaging (MRI) [1]. Clinical acceptance of a non-histopathologic diagnosis of HCC has pre-empted liver biopsy in many cases, saving patients from an invasive diagnostic procedure. However, a liver biopsy may still be required if imaging is inconclusive. Presently, the radiographic diagnosis of HCC relies heavily on the assessment of tumor contrast enhancement. Since metabolic reprogramming is considered one of the hallmarks of cancer [2], molecular imaging techniques, such as positron emission tomography (PET)/CT and chemical-shift encoded MRI might provide complementary diagnostic information related to tumor metabolism or biochemical composition. This orthogonal information could have incremental value for diagnosis in the event that conventional imaging is inconclusive, thus, preserving the non-invasive nature of the diagnostic work-up of HCC.

The study of tissue metabolomics could facilitate the search for additional imaging approaches. For example, targeted metabolomics can quantitatively profile a large variety of biologically active molecules in tissues to identify new and novel molecular imaging targets. However, targeted metabolomics typically requires that the metabolites of interest be identified a priori, which historically has limited its usefulness for biomarker discovery. Nonetheless, one important advantage of targeted metabolomics over untargeted approaches is that its results are more amenable to biological interpretation and functional profiling, potentially streamlining molecular imaging development. On contemporary platforms, targeted metabolomic analyses are currently capable of quantitatively assaying hundreds of related compounds comprising entire classes of metabolites with relatively high throughput. By comparing these targeted profiles between tumor and non-tumor tissue samples, it is possible to comprehensively evaluate class-specific biomarker signatures for HCC.

The liver is a multi-functional organ whose major biological roles include carbohydrate metabolism, bile production, protein and lipid synthesis, chemical detoxification, and vitamin and mineral storage. Because a number of these hepatic functions are known to be altered in HCC tumors, metabolites corresponding to these liver functions may be potential biomarkers of HCC. For HCC, liver-related metabolites worth pursuing biomarkers include bile acids, fatty acids, lipids, and the small-molecules associated with organelle functions and energy metabolism.

Machine learning (ML) can be applied to distinguish patterns in high-dimensional data, which can potentially lead to more accurate diagnostic predictions or classifications than traditional statistical models. Support vector machine (SVM), partial least squares discriminant analysis (PLSDA), and random forests (RF) models are well-understood ML classification approaches that have been used successfully to develop clinical diagnostics [3]. Classification signatures developed by ML can also be utilized to screen and comparatively evaluate metabolomics data as sources of biomarkers. The purpose of this study was to apply ML to comparatively evaluate four distinct liver-related metabolite classes (bile acids, free fatty acids, lipids, and small molecules) as potential sources of biomarkers to distinguish HCC from non-tumor liver tissue.

## 2. Materials and Methods

### 2.1. Patients

Between February 2012 and March 2017, 53 patients gave written informed consent as participants in an Institutional Review Board-approved clinical research study (The Queen’s Medical Center Research and Institutional Review Committee ID RA-2011-025, approved 11 May 2011) that examined tumor and corresponding liver tissue samples obtained following treatment of HCC by partial hepatectomy. Briefly, patients were eligible if they had HCC diagnosed histologically, or suspected radiographically, or had a liver mass with imaging features of primary malignancy, and were surgical candidates (i.e., diagnosed early stage HCC) with Child-Pugh score < 10. Patients were excluded if they had received prior chemotherapeutic, molecularly targeted, biological, or radiotherapeutic treatment for HCC. All tumor and non-tumor liver tissue samples were obtained intra-operatively, divided and placed separately in labeled cryovials for storage in liquid nitrogen until retrieved for analysis. Tumor status was confirmed by histopathologic review of each specimen. Retrieved samples were temporarily stored at −80 °C (Forma 8600 series ultra-low temperature freezer, Thermo-Fisher Scientific, Nashville, NC, USA) in preparation for metabolomic analysis.

### 2.2. Chemical Reagents

Methanol, acetonitrile, and formic acid were purchased from Thermo-Fisher Scientific (Optima LC-MS, Fair Lawn, NJ, USA). Ultrapure water was produced by a Mill-Q Reference system equipped with LC-MS Pak filter (Millipore, Billerica, MA, USA). The derivatization reagents, methoxyamine hydrochloride and N-methyl-trimethylsilyltrifluoroacetamide (MSTFA)were purchased from Sigma-Aldrich (St. Louis, MO, USA). Analytical grade sodium hydroxide, sodium bicarbonate, and anhydrous sodium sulfate were obtained from JT Baker Co. (Phillipsburg, NJ, USA). All of the 57 bile acid standards were obtained from Steraloids Inc. (Newport, RI, USA) and TRC Chemicals (Toronto, ON, Canada) and nine stable isotope-labeled standards were obtained from C/D/N Isotopes Inc. (Pointe-Claire, Quebec, Canada) and Steraloids Inc. (Newport, RI, USA). All other standards were commercially purchased from Sigma-Aldrich and Nu-Chek Prep (Elysian, MN, USA). A total of 145 representative compounds of different chemical classes was used for metabolomic analysis.

### 2.3. Sample Preparation and Analysis

The standards and stable isotope-labeled standards were accurately weighed and prepared in methanol at a concentration of 5.0 mM (stock solution). Further dilution was performed with a methanol/water mixture (50/50, *v*/*v*) to obtain calibration concentrations of 2000, 400, 160, 32, 12.8, 2.5, or 1 nM.

### 2.4. Bile Acid Profiling (UPLC-MS)

The preparation of samples for bile acid profiling was based on modification of our published methods [4,5,6]. Briefly, liver tissue samples were accurately weighed (~20 mg) and then homogenized with 50 μL of water using a Bullet Blender Tissue Homogenizer (Next Advance, Inc., Averill Park, NY, USA). An aliquot of 150 μL of acetonitrile containing nine internal standards was added, and the extraction was performed using the homogenizer. After centrifugation, each 50 μL of the supernatant was transferred to a 96-well plate.

Bile acids were quantitated using ultra-performance liquid chromatography coupled to tandem mass spectrometry (UPLC-MS/MS, ACQUITY UPLC-Xevo TQ-S, Waters Corp., Milford, MA, USA) using an ACQUITY UPLC BEH C18 1.7 µM Vanguard pre-column (2.1 × 5 mm) and ACQUITY UPLC BEH C18 1.7 µM analytical column (2.1 × 100 mm) with the following optimized settings: Column temperature 45 °C, sample manager temperature 10 °C, mobile phases: A = water with formic acid (pH = 3.25), B = acetonitrile /methanol (95:5); gradient 0–1 min (5% B), 1–5 min (5–25% B), 5–15.5 min (25–40% B), 15.5–17.5 min (40–95% B), 17.5–19 min (95% B), 19–19.5 min (95.5% B), 19.6–21 min (5% B); flow rate 0.45 mL/min; capillary kv 1.2 (ESI negative), source temperature 150 °C; desolvation temperature 550 °C; desolvation gas flow 1200 L/h.

The data were collected with multiple reaction monitor (MRM) (Supplementary Information 1) using optimized settings from QuanOptimize application manager (Waters, Milford, MA, USA). The calibration curve and the corresponding regression coefficients were obtained by internal standard adjustment (Supplementary Information 1). All bile acids were found to be linear over the measured range.

### 2.5. Fatty Acid and Small Molecule Profiling (GC-TOFMS)

Sample preparation for gas chromatography-time of flight mass spectrometry (GC-TOFMS) analysis of free fatty acids and other small molecules was performed according to our published methods [7]. GC analysis was carried out on a GC-TOFMS system (LECO Corp., St. Joseph, MI, USA) using a Rxi-5 MS (Crossbond × 5% diphenyl/95% dimethyl polysiloxane) column with the following GC instrument settings: Oven program 80 °C (2 min), 80–140 °C (10 °C/min), 140–210 °C (4 °C/min), 210–240 °C (10 °C/min), 240–290 °C (25 °C/min), 290 °C (4.5 min); injection volume 1 µL; inlet temperature 270 °C; inlet mode: Splitless; carrier gas: Helium (99.9999%), flow rate 1.0 mL/min constant; transfer interface temperature 270 °C. TOFMS was performed using the following optimized settings: Electron impact ionization mode; electron energy −70 V; detector voltage −1450 V; source temperature 220 °C; solvent delay 4.1 min; acquisition rate 25 spectra/second; mass range 50–500 Da.

### 2.6. Lipid Profiling (UPLC-MS)

For lipid profiling, 20 µL aliquots of the supernatant were added to a 96-well plate. After drying under nitrogen, 300 µL of a 5 mM solution of ammonium acetate in methanol was added, and the plate was gently shaken at room temperature for 30 min. Sample extracts were filtered through 0.45 µm membrane of the kit plate and each 20 µL aliquot was further diluted with 380 µL of methanol with 5 mM ammonium acetate based on our published methods [8]. Targeted metabolite analysis of 140 lipids was then performed using an ACQUITY UPLC-Xevo TQ-S (Waters Corp., Milford, MA, USA). Each 10 µL volume of sample was directly injected into the mass spectrometer. A 5 mM solution of ammonium acetate in methanol was used as eluant at an increasing flow rate (30 to 200 µL/minute within 3 min).

### 2.7. Data Processing/Analysis

Raw data from UPLC—MS/MS was processed using the TargetLynx application manager (Waters Corp., Milford, MA, USA) to obtain calibration equations and quantitative concentrations of each metabolite in the samples. Raw data from GC-TOFMS analysis were exported to the ChromaTOF software (v4.50, Leco Co., CA, USA) for baseline correction, smoothing, noise reduction, deconvolution, library searching, and area calculation. For the GC−TOFMS generated data, identification was processed by comparing the mass fragments and the retention time with our in-house library or the mass fragments with NIST 05 Standard mass spectral databases in NIST MS search 2.0 (NIST, Gaithersburg, MD, USA) software using a similarity of more than 70%. The detected metabolites from GC-TOFMS were annotated and combined using automated mass spectral data processing software [9]. Samples or compounds with significant loss of data (10% of data was missing) were excluded from further analysis. These quantification protocols using authentic standards resulted in quantitative profiles for the following four classes of metabolites: Bile acids (BA, 42 metabolites), phospholipids (lipids, 109 metabolites), and other small molecules including free fatty acids (FFA) (128 metabolites total).

### 2.8. Biomarker and Statistical Analysis

Biomarker discovery and evaluation were carried out using MetaboAnalyst (McGill University, Montreal, CA, USA), accessed via the metaboanalyst.ca web-portal v4.0 or implemented locally in R using the MetaboAnalystR 2.0 package [10,11,12]. Missing value imputation was performed by the K-nearest neighbor (KNN) algorithm. The dataset was normalized, transformed, and scaled by row-wise quantile normalization, log transformation, and mean centering (Supplementary Figure S1).

Multivariate receiver operating characteristic (ROC) and area under the ROC curve (AUC) values were calculated to test the hypothesis that specific metabolite classes differed in classification performance for distinguishing between the tumor and non-tumor samples. For this pilot study, a sample size of 41 test and 41 control samples provided 0.80 power with 2-sided type 1 error rate of 0.05 for detecting a 0.2 AUC difference in the expected range of AUC values. The 95% confidence intervals (CI) for AUC were calculated. The ML classification methods used for automated feature identification were SVM and random forests. For the SVM classification method, features were ranked by their relative contribution to correct classification based on cross-validation error rates [13]. For the random forests classification method, features were ranked based on their mean decreases in accuracy across permutations [14]. Corresponding ROC curves were generated by Monte-Carlo cross-validation using balanced subsampling as implemented by MetaboAnalyst.

## 3. Results

Five patients did not contribute any adjacent liver tissue samples, due to sample scarcity, and five patients did not contribute tumor tissue samples, due to tumoral necrosis. The patient clinical characteristics are summarized in Table 1. One sample (i.e., row) was excluded from further analysis, due to > 10% missing data. The following compounds (i.e., columns) were also excluded, due to missing values (% missing shown): Alpha-hydroxyisobutyric acid (17.7%), pimelic acid (32.3%), 3-methyladipic acid (21.9%), isovaleric acid (31.3%), PC aa C36:0 (40.6%), SM C20:2 (26.0%). Thus, the final data set comprised 96 tissue samples (48 tumor and 48 adjacent liver, unpaired) with concentration values corresponding to 76 small molecules, 42 bile acids, 47 free fatty acids, and 107 lipid compounds.

**Figure 1:**
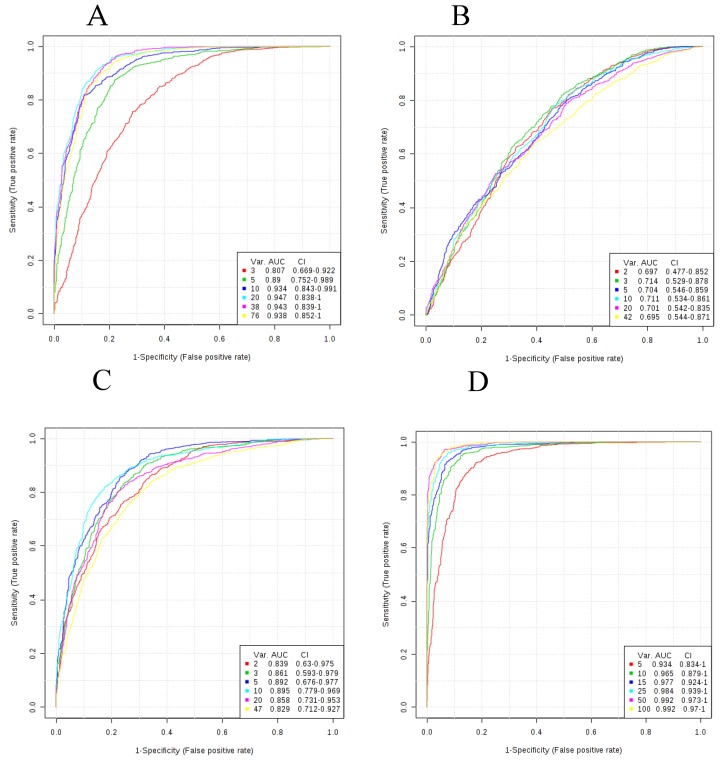

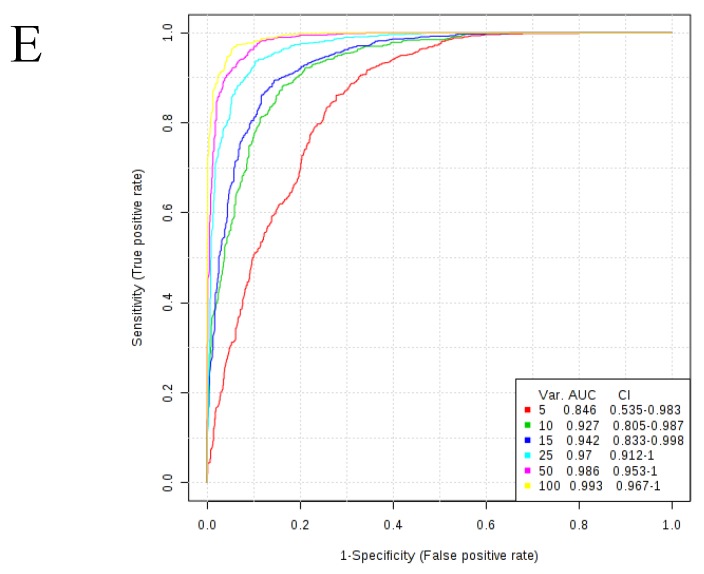
ROC curves for SVM signatures based on each of the metabolite classes. Plots reflect average performance across all Monte Carlo cross validation runs. (**A**) Small molecules; (**B**) Bile acids; (**C**) Free fatty acids; (**D**) Lipids; (**E**) All metabolites. All 95 percent confidence intervals (CIs) were computed. The signature size corresponding to each colored curve is shown under the “Var” heading shown in the key for each plot.

**Figure 2:**
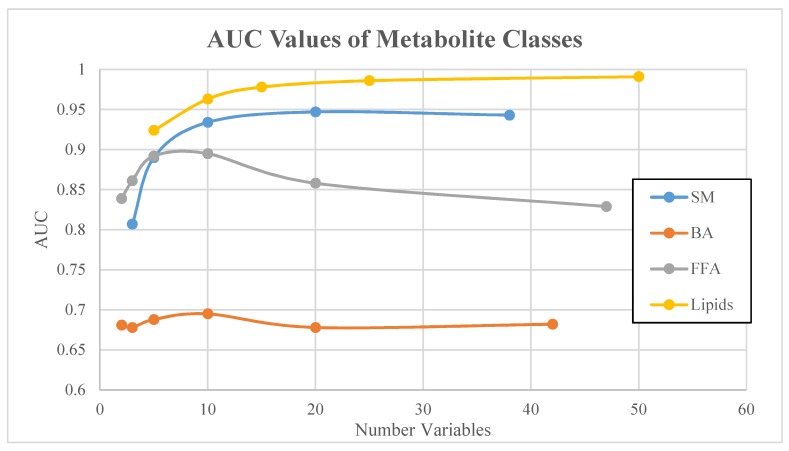
AUC values of all four metabolite classes as a function of number of variables.

**Figure 3:**
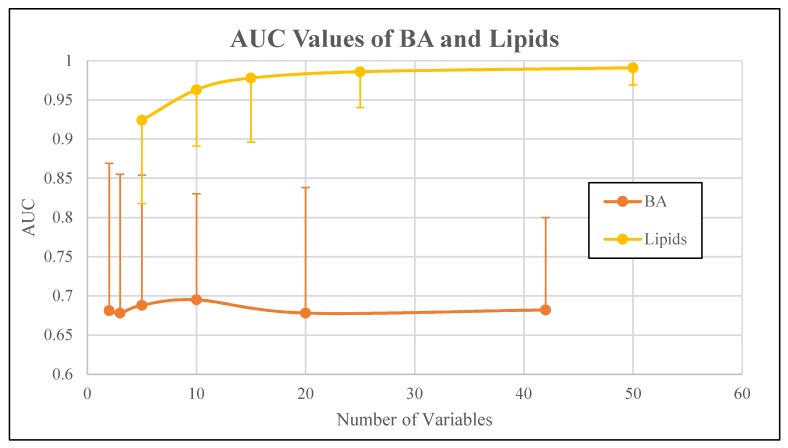
AUC values of bile acids (BA) and lipids metabolite classes. Vertical lines correspond to 95% confidence intervals.

AUC values for SVM-derived metabolomic signatures comprised of 3 to 50 signature variables (i.e., metabolites) from each class are shown in Table 2. The ROC curves corresponding to metabolite class-based signatures derived by SVM are displayed in Figure 1. A plateau in overall classification performance of the SVM-based signatures was observed at approximately 10 metabolites for each of the metabolite classes (Figure 2). Taking into account the number of signature variables, the lipid signatures were associated with the highest AUC values, followed by signatures derived from small molecules, FFA, and BA metabolites. Differences in AUC were not significant in most cases, with the exception of significant differences in AUC values between lipid signatures and bile acid signatures (Figure 3). Metabolite signatures derived by random forests performed similarly (Supplementary Figures S2–S4). Lipid signatures were associated with the highest AUC values followed by signatures derived from SM, FFA, and BA metabolites (Supplementary Table S1). The compounds that comprised the 10-metabolite signatures derived by SVM and random forests are listed in Table 3 and Supplementary Table S2, respectively. Individual metabolite fold changes and their associated false discovery rates are provided in Supplementary Information 2.

## 4. Discussion

Currently, HCC is one of the few cancers in which tumor biopsy is not clinically required to establish the diagnosis. In appropriately selected patients, contrast-enhanced imaging studies have proven sufficient for confidently diagnosing HCC [1]. However, the existing radiographic approaches do not work for all patients [15]. Metabolic alterations constitute an alternative and orthogonal set of diagnostic targets for which novel imaging methods are being developed or have been developed. In this study, ML was applied in the manner used for metabolomics signature discovery, but with the primary objective to compare different classes of metabolites as potential sources of HCC biomarkers. We believe results from this comparison between classes of metabolites may be useful for prioritizing which metabolic pathways to pursue further for molecular imaging of HCC. Together with other information obtained from histopathologic, genomic, and transcriptomic analyses [16,17], this metabolomic data may help to inform the development of new imaging strategies for HCC. It is hoped that the success rate and utilization of non-histopathologic diagnostic algorithms will increase through the development of new tumor imaging modalities.

To limit bias in our metabolomic comparisons, we applied SVM and random forests, two well-developed machine learning algorithms, in systematic fashion [18]. Classification performance was examined over a range of signature size, and a comparison of AUC values was used to determine which metabolite class comprised the most promising source of HCC biomarkers. While differences in AUC values were not significant in most cases, lipid-based signatures were consistently associated with the highest AUC values across a range of signature size. A comparison of signatures derived using random forests closely mirrored the results produced by SVM-based signatures. These findings serve as encouragement to further pursue the development of HCC biomarkers derived from phospholipid pathways.

Metabolic reprogramming is considered one of the hallmarks of cancer [2]. Multiple alterations in BA, lipid, FFA, and energy molecule metabolism have been observed in HCC [19,20,21,22,23]. Because of these myriad metabolic derangements in HCC, there was no a priori hypothesis as to which class of metabolites would perform the best. Alterations in the cellular profiles of small molecular metabolites, such as glucose, glycerol 3- and 2-phosphate, malate, alanine, and myo-inositol have been observed in other metabolomic studies of HCC [19]. Significant alterations in nutrient uptake by cancer cells have also been noted, and may contribute to the changes in the cellular concentrations of energy metabolites, amino acids, and other small molecules [22]. These alterations may also affect the intracellular synthesis of specific nutrients relevant to cancer metabolism, such as glutamine [22]. In our study, these metabolites were represented in the signatures derived from small molecules.

Several of the metabolites which comprised our small molecule signatures, including malate, pyruvate, and creatine, can already be measured in vivo by molecular imaging techniques, such as magnetic resonance spectroscopy or hyperpolarized MRI [24,25]. In our study, malic acid alone was associated with an AUC of 0.83 (Table 3). Malate synthesis can be imaged in vivo by MRI using the hyperpolarized contrast agent ^13^C-fumarate, although its usage has mainly been to monitor cellular necrosis [26]. We are unaware of studies that have evaluated hyperpolarized ^13^C-fumarate as an imaging agent for HCC. Hyperpolarized ^13^C-pyruvate has been used with MRI to detect HCC in rodent models [27]. These pilot results support efforts to develop hyperpolarized contrast agents for imaging HCC further.

Recognition that fatty acid metabolism is dramatically altered in cancer cells has increased in recent years. These alterations include changes in both fatty acid synthesis, as well as degradation/oxidation, and several potential anti-cancer drugs targeting these pathways are currently in development [28]. In HCC, there appears to be coordinated activation of fatty acid synthesis and lipogenesis, possibly as a result of AKT-mTOR signaling pathway activation [29]. PET imaging using the true tracer ^11^C-acetate has been proposed as a means to image fatty acid synthesis in vivo [30]. While PET using ^11^C-acetate has been used to image de novo lipogenesis in tumors [30], one study recently found that tissue uptake of this tracer does not correlate with fatty acid synthase expression [31]. A number of novel PET probes for imaging fatty acid oxidation have also been developed [32,33,34]. In addition to free fatty acid synthesis, fatty acid uptake may also be altered in cancer cells [35]. This is consistent with the finding of linolenic acid in our FFA signatures, since it is an essential fatty acid that must be gotten from the diet. The finding of palmitelaidic acid in our FFA signatures is also intriguing, since it is a trans fatty acid whose major dietary sources are hydrogenated vegetable oils and dairy fats. Trans fatty acid-rich diets have been shown to increase liver tumorigenesis in mouse models [36]. Recent studies have also suggested that lipid desaturation (i.e., formation of double bonds in the fatty acyl chains) also occurs more frequently in cancer cells, providing additional opportunities to interfere with cancer cell fatty acid metabolism by targeting the desaturase enzymes [37,38]. Our results suggest that the proportions of saturated, mono-unsaturated, and poly-unsaturated fatty acids differ between HCC and liver tissue. MRI-based techniques for profiling the composition of fatty acids are emerging [39]. Our results support further investigation of fatty acid profiles as imaging biomarkers for HCC.

Enterohepatic circulation of bile acids may play an important role in carcinogenesis, and possibly involves crosstalk with the gut microbiota [23]. The interaction between bile acids and gut microbiome may also produce alterations in the bile acid profiles of liver tissues [40], supporting the hypothesis that metabolomic signatures for HCC can potentially be derived from profiling of bile acids in these tissues. There may also be alterations in cellular export of bile salts in HCC, and bile salt export pump (BSEP) has been proposed as an immunohistopathologic marker of HCC [41]. Despite these findings supporting bile acids as tissue biomarkers for HCC, our results suggest that bile acids may not be as promising a source of HCC tissue biomarkers as other classes of metabolites. However, bile acid profiles obtained from blood or other body fluids could have diagnostic value. Our previous work has indeed shown that bile acids are differentially expressed in the serum or urine of patients with HCC [42]. However, levels of several of the bile acids identified are potentially influenced by the underlying severity of chronic liver disease (CLD), with abnormal levels of GCA, TCA, CDCA, and glycochenodeoxycholic acid being associated with cirrhosis and hepatitis [42]. Although all patients in the present study had underlying liver disease of milder severity (Child-Pugh Score < 10), it remains possible that even mild liver dysfunction may have influenced tissue bile acid concentrations. Further research on the potential of tissue bile acid profiles as biomarkers of CLD is needed.

In most tissues, the majority of lipids are in the form of phospholipids. The phospholipid signatures that best discriminated HCC from non-tumor liver tissue in our study included several different species of phosphatidylcholines. PET imaging using ^18^F-fluorocholine is an imaging biomarker of phosphotidylcholine synthesis that is currently used for clinical detection of HCC in some regions [43,44] It has been shown to be superior to PET imaging of glucose metabolism with ^18^F-fluoro-deoxy-D-glucose (FDG) for the detection of HCC, implying that lipogenesis is more salient than glycolysis as a metabolic feature of HCC [43,45,46]. Other studies have identified lipogenic networks characterized by specific lipid metabolites as being associated with HCC progression and survival [20]. The Wnt/beta-catenin pathway has been implicated in hepatocarcinogenesis [47], and mutations causing activation of beta-catenin have been associated with increased tumor phospholipid biosynthesis and uptake of ^18^F-fluorocholine in HCC [44]. Beta-catenin activation has also been associated with increased fatty acid oxidation in HCC [48]. Imaging of lipid metabolism may therefore have the potential to discriminate specific molecular sub-types of HCC [17,44].

This study was limited in that we did not pursue further testing and validation with independent datasets. However, our goal was to compare different classes of metabolites as potential sources of biomarkers and not to develop a specific tissue metabolomic signature for HCC. The results of this study were intended to encourage further development of new biomarkers for HCC, including those which can be measured non-invasively using different molecular imaging techniques. Another limitation worth noting is that targeted metabolomics restricts the scope of analysis to compounds defined a priori. While very abundant or biologically relevant compounds from each metabolite class were selected for this study, not all the variance associated with each class may have been captured. This study also used 2 ML approaches to compare the metabolite classes in a relatively unbiased manner. There is no universal agreement on the optimal classification method for metabolomics and the possibility of overfitting by ML algorithms should not be ignored. However, SVM and random forests classification methods have been found more resilient to noise and overfitting than other methods applied to metabolomics data [49]. The slight improvement in classification performance from signatures derived from random forests over SVM mirrored results seen with other array datasets [18].

## 5. Conclusions

Metabolomic profiles composed of bile acids, free fatty acids, phospholipids, and metabolically-active small molecules were systematically analyzed to discover potential signatures to discriminate HCC from adjacent liver tissue. While extensive testing and validation of these signatures would be necessary to substantiate their performance as diagnostic tissue biomarkers, the composition of these signatures and relative performance based on AUC can immediately inform the development of next-generation molecular imaging techniques for detecting HCC. Through comparisons of the classification performance of metabolite signatures discovered using SVM and random forests ML algorithms, phospholipids were found to be the class of metabolites that showed the most promise in this pilot study for distinguishing tumor and non-tumor samples from patients with HCC. While rudimentary molecular imaging biomarkers for phospholipid metabolic reprogramming already exist, these results should encourage further development and refinement of lipid molecular imaging with the specific goal of improving the overall accuracy of imaging-based diagnostic algorithms for HCC.

## Figures and Tables

**Table 1:** Patient clinical characteristics.

	Females (*n* = 12)	Males (*n* = 36)	*p*-Value
Age, years, mean (SD)	61.58 (13.14)	62.78 (10.32)	0.747
HBV Infected, number (%)	3 (25.0)	10 (27.8)	1.00
HCV Infected, number (%)	3 (25.0)	18 (50.0)	0.240
Tumor Grade, number (%)			0.259
G1	2 (16.7)	2 (5.6)	
G2	3 (25.0)	20 (55.6)	
G3	5 (41.7)	11 (30.6)	
G4	2 (16.7)	3 (8.3)	
AFP level, ng/dL, mean (SD)	2409.2 (3897.3)	886.8 (3281.8)	0.191
Child-Pugh Score, mean (SD)	5.50 (0.67)	5.53 (1.08)	0.934
MELD Score, mean (SD)	8.00 (1.41)	8.82 (2.52)	0.296

**Table 2:** Differences in area under the receiver operating characteristic curve (AUC) values for support vector machine (SVM)-derived metabolomic signatures of a varying number of metabolite variables in the signature.

Metabolite Class	Number of Metabolites in Signature	Area Under Curve (AUC)	Lower Bound 95% CI	Upper Bound 95% CI
Small molecules	3	0.807	0.669	0.922
	5	0.89	0.752	0.989
	10	0.934	0.843	0.991
	20	0.947	0.838	1
	38	0.943	0.839	1
Bile acids	2	0.681	0.44	0.869
	3	0.678	0.448	0.855
	5	0.688	0.446	0.854
	10	0.695	0.51	0.83
	20	0.678	0.498	0.838
	42	0.682	0.514	0.8
Free fatty acids	2	0.839	0.63	0.975
	3	0.861	0.593	0.979
	5	0.892	0.676	0.977
	10	0.895	0.779	0.969
	20	0.858	0.731	0.953
	47	0.829	0.712	0.927
Lipids	5	0.924	0.818	0.979
	10	0.963	0.891	0.999
	15	0.978	0.896	1
	25	0.986	0.94	1
	50	0.991	0.969	1

**Table 3:** The compounds comprising the SVM-derived 10-metabolite signatures for four different metabolic classes (small molecules, free fatty acids, bile acids, and phospholipids). Fold change, false discovery rate (FDR), along with univariate *p*-value and area under the receiver operating characteristic curve (AUC) are shown for each metabolite univariate.

	**Small Molecules**				
**Rank**	**Metabolite**	**Fold Change**	**AUC**	***p*-Value**	**FDR**
1	Dimethylglycine	0.63	0.74	1.59 × 10^−05^	2.41 × 10^−04^
2	6-Phosphogluconic acid	0.518	0.689	1.51 × 10^−03^	8.18 × 10^−03^
3	Pyruvic acid	2.131	0.755	3.75 × 10^−06^	7.13 × 10^−05^
4	D-2-Hydroxyglutaric acid	2.273	0.721	1.30 × 10^−03^	7.58 × 10^−03^
5	L-alpha-aminobutyric acid	0.652	0.692	2.22 × 10^−03^	9.92 × 10^−03^
6	Glycerophosphocholine	0.764	0.696	1.03 × 10^−03^	7.11 × 10^−03^
7	Glyceric acid	1.291	0.144	4.66 × 10^−01^	6.65 × 10^−01^
8	N-Acetylornithine	1.223	0.22	9.46 × 10^−02^	2.24 × 10^−01^
9	Creatine	0.644	0.771	9.07 × 10^−07^	2.30 × 10^−05^
10	Malic acid	0.478	0.834	3.27 × 10^−08^	2.49 × 10^−06^
	**Free Fatty Acids**				
**Rank**	**Metabolite**	**Fold Change**	**AUC**	***p*-Value**	**FDR**
1	Alpha-Linolenic acid	0.364	0.783	2.83 × 10^−07^	1.33 × 10^−05^
2	Palmitelaidic acid	1.749	0.738	1.84 × 10^−05^	3.61 × 10^−04^
3	Butyric acid	1.79	0.72	3.07 × 10^−05^	3.61 × 10^−04^
4	3-Hydroxybutyric acid	0.626	0.571	1.08 × 10^−01^	2.55 × 10^−01^
5	10Z-Heptadecenoic acid	1.395	0.689	1.02 × 10^−03^	9.56 × 10^−03^
6	Gamma-Linolenic acid	0.989	0.552	5.25 × 10^−01^	7.47 × 10^−01^
7	8,11,14-Eicosatrienoic acid	1.853	0.736	2.47 × 10^−05^	3.61 × 10^−04^
8	Valeric acid	1.478	0.609	7.48 × 10^−02^	1.95 × 10^−01^
9	Undecanoic acid	0.837	0.6	5.93 × 10^−02^	1.86 × 10^−01^
10	Docosahexaenoic acid	1.105	0.527	8.73 × 10^−01^	9.33 × 10^−01^
	**Bile Acids**				
**Rank**	**Metabolite**	**Fold Change**	**AUC**	***p*-Value**	**FDR**
1	Chenodeoxycholic acid	4.57	0.752	2.93 × 10^−05^	6.16 × 10^−04^
2	Glycholic acid	0.627	0.735	1.67 × 10^−05^	6.16 × 10^−04^
3	Dihydroxycholestanoic acid	1.339	0.544	5.90 × 10^−01^	8.54 × 10^−01^
4	7-ketolithocholic acid	1.603	0.608	3.12 × 10^−02^	1.09 × 10^−01^
5	Cholestenoic acid	1.057	0.631	3.98 × 10^−01^	7.36 × 10^−01^
6	6,7-diketolithocholic acid	0.91	0.577	1.49 × 10^−01^	4.48 × 10^−01^
7	Deoxycholic acid	5.751	0.578	3.12 × 10^−02^	1.09 × 10^−01^
8	Tauroursodeoxycholic acid	1.552	0.681	8.26 × 10^−01^	9.84 × 10^−01^
9	Chenodeoxycholic acid 24-glucuronide	1.388	0.553	7.96 × 10^−01^	9.84 × 10^−01^
10	Lithocholic acid 3-sulfate	1.177	0.614	2.02 × 10^−02^	1.03 × 10^−01^
	**Phospholipids**				
**Rank**	**Metabolite**	**Fold Change**	**AUC**	***p*-Value**	**FDR**
1	PC aa C26:0	0.603	0.782	6.62 × 10^−07^	6.44 × 10^−06^
2	PC ae C34:0	3.037	0.873	5.43 × 10^−12^	2.90 × 10^−10^
3	PC ae C34:2	0.836	0.658	3.93 × 10^−04^	1.31 × 10^−03^
4	PC aa C32:0	1.312	0.653	1.46 × 10^−02^	2.95 × 10^−02^
5	PC aa C38:6	0.473	0.918	1.37 × 10^−13^	1.46 × 10^−11^
6	PC aa C42:2	1.386	0.818	4.78 × 10^−06^	2.56 × 10^−05^
7	PC aa C40:5	0.808	0.722	1.80 × 10^−04^	6.41 × 10^−04^
8	PC aa C34:3	0.687	0.741	8.23 × 10^−06^	4.19 × 10^−05^
9	PC ae C32:2	1.673	0.703	1.72 × 10^−04^	6.33 × 10^−04^
10	PC ae C44:3	1.49	0.803	3.13 × 10^−08^	5.58 × 10^−07^

## Supplementary Materials

The following are available online at https://www.mdpi.com/2075-4418/9/4/167/s1.
