# Supplementary material for: Phospholipids are A Potentially Important Source of Tissue Biomarkers for Hepatocellular Carcinoma: Results of a Pilot Study Involving Targeted Metabolomics
Source: Diagnostics (Basel). 2019 Oct 29;9(4):167. doi: 10.3390/diagnostics9040167 (PMC6963224; doi:10.3390/diagnostics9040167)
Supplement: Supplementary file 1 [file diagnostics-09-00167-s001.zip › diagnostics-611252 suppl for final/Supplementary Files_final/Supplementary Tables and Figures.pdf]

Supplementary Materials

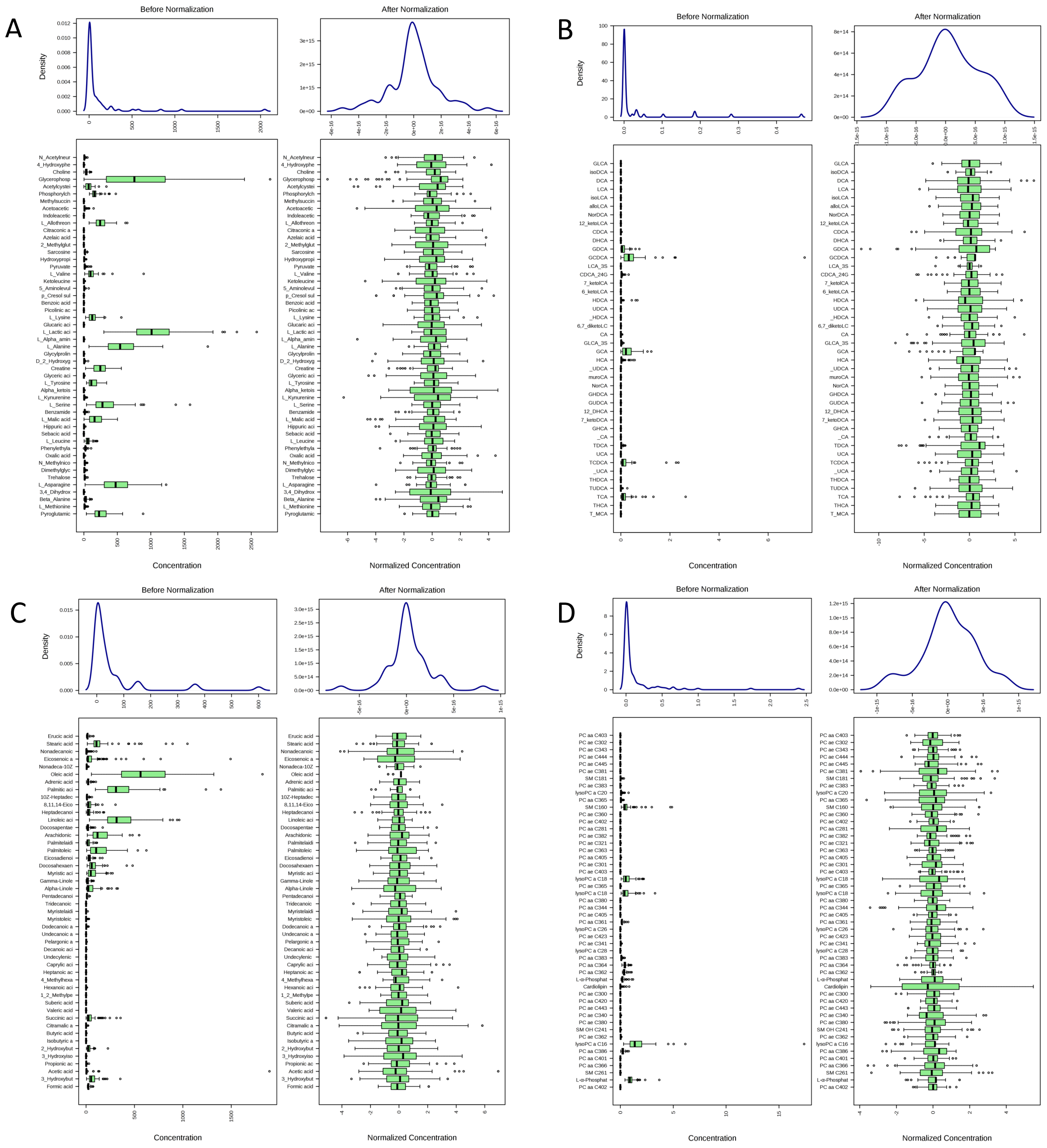

Supplementary Figure 1. Box plots and kernel density plots before and after normalization. The density plots are based on all samples. (A) Small molecules; (B) Bile acids; (C) Free fatty acids; (D) Lipids.

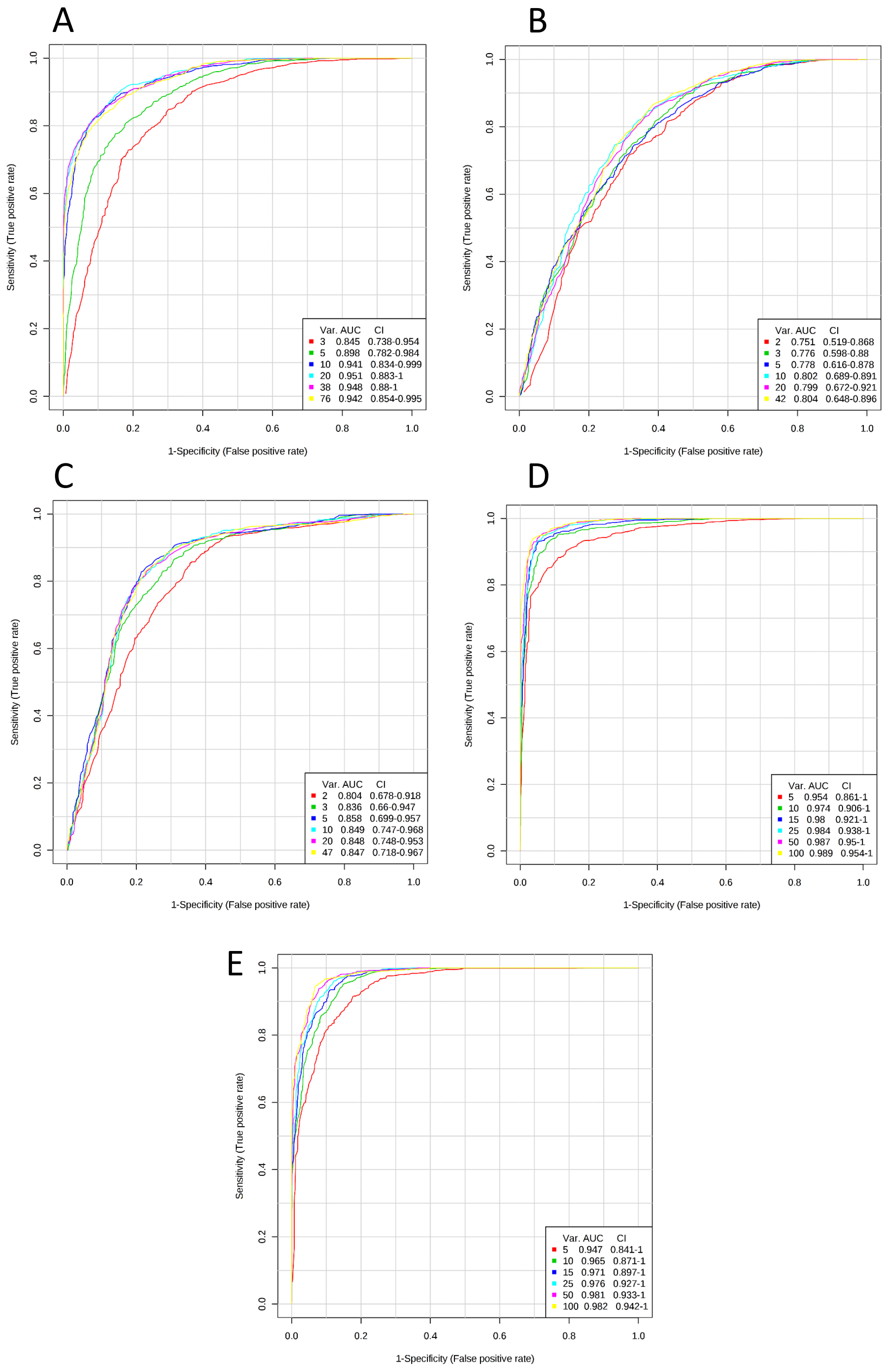

Supplementary Figure 2. ROC curves for random forests signatures based on the different metabolite classes. Plots reflect average performance across all Monte Carlo cross validation runs. (A) Small molecules; (B) Bile acids; (C) Free fatty acids; (D) Lipids; (E) All metabolites. All 95% confidence intervals (CIs) were computed. The signature size corresponding to each curve is shown under the "Var" heading in the key for each plot.

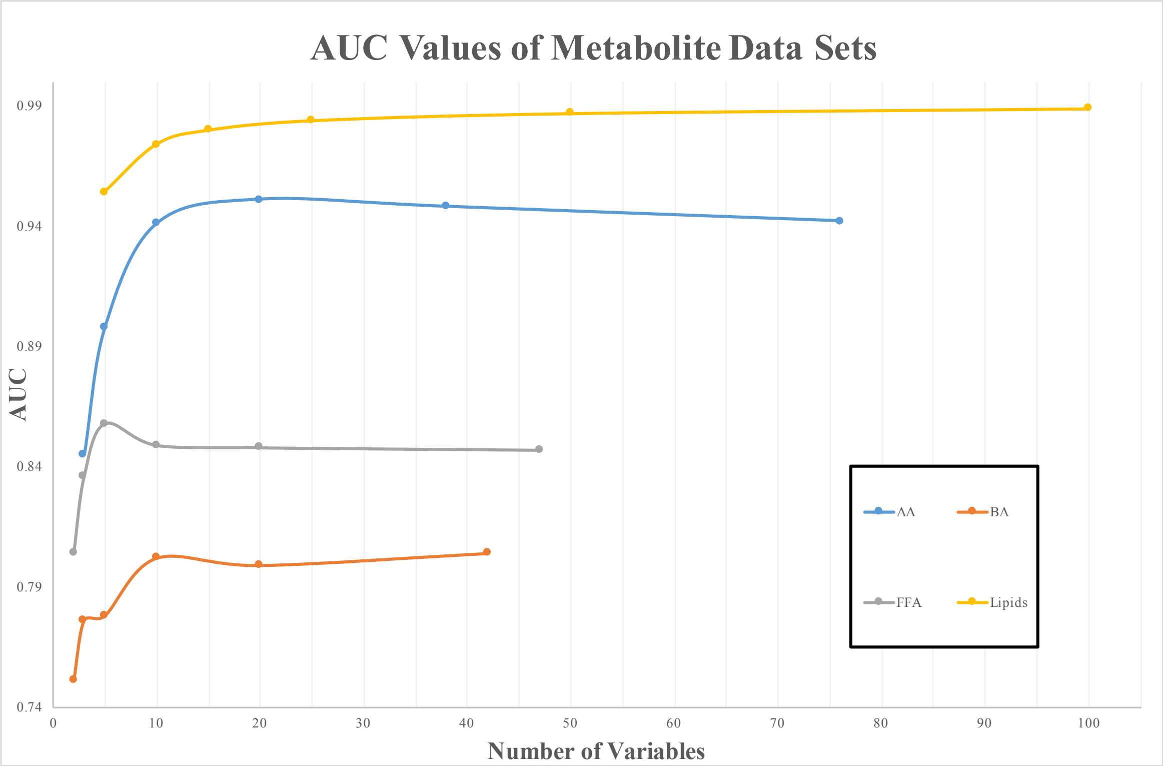

Supplementary Figure 3. AUC values for random forest based signatures of all four metabolite classes.

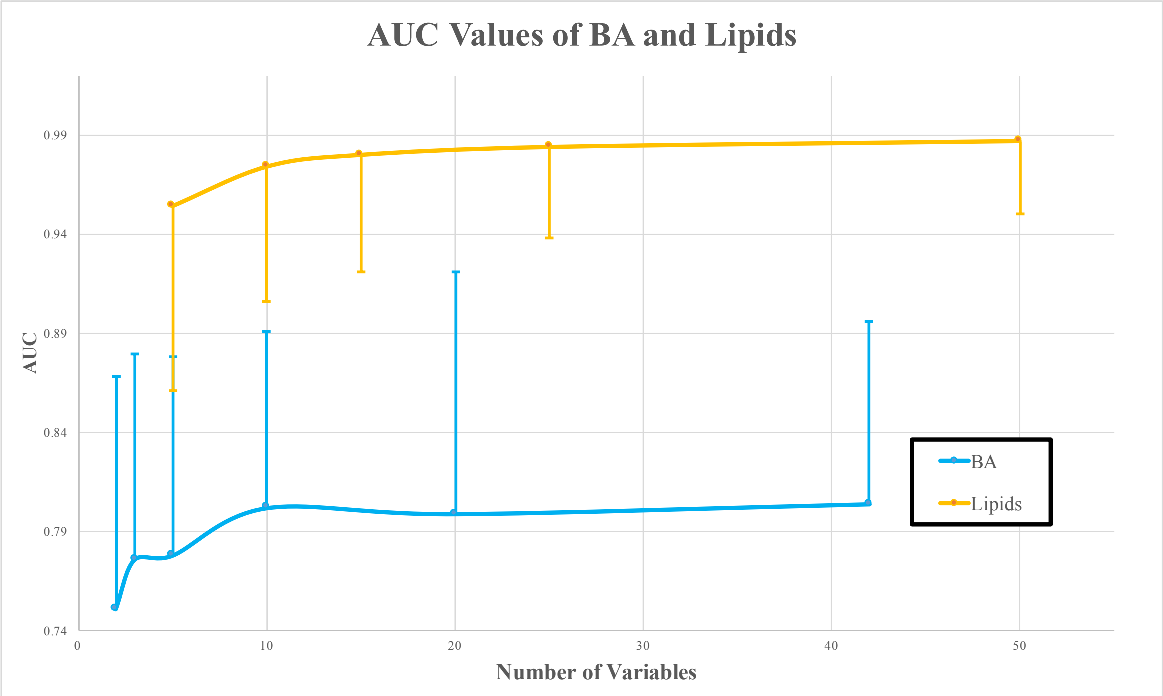

Supplementary Figure 4. AUC values for random forests based signatures for BA and lipids metabolite classes. Horizontal bars represent 95% confidence intervals for AUC.

Supplementary Table 1: Differences in AUC values for random forests-derived metabolomic signatures of varying number of metabolite variables in the signature.

**Metabolite class**	**Number of Variables**	**Area Under Curve (AUC)**	**Lower bound 95% CI**	**Upper bound 95% CI**
Small molecules	3	0.845	0.738	0.954
	5	0.898	0.782	0.984
	10	0.941	0.834	0.999
	20	0.951	0.883	1
	38	0.948	0.880	1
Bile acids	2	0.751	0.519	0.868
	3	0.776	0.598	0.880
	5	0.778	0.616	0.878
	10	0.802	0.689	0.891
	20	0.799	0.672	0.921
	42	0.804	0.648	0.896
Free fatty acids	2	0.804	0.678	0.918
	3	0.836	0.660	0.947
	5	0.858	0.699	0.957
	10	0.849	0.747	0.968
	20	0.848	0.748	0.953
	47	0.847	0.718	0.967
Phospholipids	5	0.954	0.861	1
	10	0.974	0.906	1
	15	0.980	0.921	1
	25	0.984	0.938	1
	50	0.987	0.950	1

Supplementary Table 2: Compounds comprising the random forests-derived 10-metabolites for 4 different metabolic classes (small molecules, free fatty acids, bile acids, and phospholipids). Fold change, false discovery rate (FDR), along with univariate p-value and area under the receiver operating characteristic curve (AUC) are shown for each metabolite univariate.

Small molecules

Rank	Metabolite	Fold Change	AUC	p-value	FDR
1	Malic acid	0.478	0.834	3.27E-08	2.48E-06
2	Maleic acid	0.487	0.789	3.46E-07	1.31E-05
3	D-2-Hydroxyglutaric acid	2.27	0.721	1.30E-03	7.60E-03
4	Pyruvic acid	2.131	0.755	3.75E-06	7.13E-05
5	Creatine	0.644	0.771	9.07E-07	2.30E-05
6	Dimethylglycine	0.63	0.74	1.59E-05	2.41E-04
7	L-Histidine	1.464	0.68	7.87E-04	5.98E-03
8	L-alpha-aminobutyric acid	0.652	0.692	2.22E-03	9.92E-03
9	L-Tryptophan	1.839	0.728	5.18E-05	6.56E-04
10	Glycerophosphocholine	0.764	0.696	1.00E-03	7.10E-03

Free fatty acids

Rank	Metabolite	Fold Change	AUC	p-value	FDR
1	a-Linolenic acid	0.363	0.783	2.83E-07	1.33E-05
2	8,11,14-Eicosatrienoic acid	1.853	0.736	2.47E-05	3.61E-04
3	Palmitelaidic acid	1.749	0.738	1.84E-05	3.61E-04
4	10Z-Heptadecenoic acid	1.395	0.689	1.02E-03	9.56E-03
5	Butyric acid	1.79	0.72	3.07E-05	3.61E-04
6	Linoleic acid	0.886	0.633	1.18E-01	2.63E-01
7	10Z-Nonadecenoic acid	1.188	0.629	4.91E-02	1.77E-01
8	Myristoleic acid	2.462	0.61	6.89E-02	1.90E-01
9	Propionic acid	0.619	0.682	2.95E-03	2.31E-02
10	gamma-Linolenic acid	0.886	0.552	1.18E-01	2.63E-01

Bile acids

Rank	Metabolite	Fold Change	AUC	p-value	FDR
1	Glycholic acid	0.627	0.735	1.67E-05	6.16E-04
2	Chenodeoxycholic acid	4.57	0.752	2.93E-05	6.16E-04
3	Taurocholic acid	0.86	0.554	1.35E-02	1.03E-01
4	Cholic acid	3.057	0.631	2.44E-02	1.03E-01
5	Taurodeoxycholic acid	0.587	0.681	2.32E-03	3.25E-02
6	Hyodeoxycholic acid	3.7	0.615	2.18E-02	1.03E-01
7	Hyocholic acid	2.889	0.62	1.55E-02	1.03E-01
8	T-muricholic acid	1.057	0.543	3.98E-01	7.36E-01
9	Lithocholic acid -3sulfate	1.176	0.614	2.02E-02	1.03E-01
10	Glycodeoxycholic acid	0.628	0.663	3.40E-03	3.57E-02

Phospholipids

Rank	Metabolite	Fold Change	AUC	p-value	FDR
1	PC aa C38:6	0.473	0.918	1.37E-13	1.46E-11
2	PC ae C34:0	3.037	0.873	5.43E-12	2.90E-10
3	PC aa C42:2	1.386	0.818	4.78E-06	2.56E-05
4	PC ae C40:6	0.606	0.809	2.32E-08	4.96E-07
5	PC aa C34:3	0.687	0.741	8.23E-06	4.19E-05
6	PC aa C28:1	0.645	0.756	1.08E-06	8.51E-06
7	PC aa C32:1	1.947	0.763	9.25E-08	1.41E-06
8	PC ae C36:2	0.899	0.79	3.21E-04	1.11E-03
9	PC ae C44:6	0.807	0.709	1.77E-03	4.20E-03
10	PC aa C38:5	0.857	0.836	1.17E-02	2.40E-02
